# *In vitro* maturation with letrozole priming: Can it be a solution for patients with cancerophobia? A pilot study

**DOI:** 10.4274/tjod.galenos.2020.79446

**Published:** 2020-12-10

**Authors:** Şafak Hatırnaz, Ebru Saynur Hatırnaz, Alper Başbuğ, Mine Kanat Pektaş, Onur Erol, Michael Dahan, Seang Tan

**Affiliations:** 1 Medicana Samsun International Hospital, Samsun, Turkey; 2 McGill University Faculty of Medicine, Department of Obstetrics and Gynecology, Montreal, Quebec, Canada; 3 Düzce University Faculty of Medicine, Department of Obstetrics and Gynecology, Düzce, Turkey; 4 Afyon Health Sciences University Hospital, Clinic of Obstetrics and Gynecology, Afyonkarahisar, Turkey; 5 Antalya Training and Research Hospital, Clinic of Obstetrics and Gynecology, Antalya, Turkey; 6 Originelle Women’s Health and Fertility Center, Montreal, Quebec, Canada

**Keywords:** Cancerphobia, in vitro maturation techniques, letrozole, oocytes, Ovarian Hyperstimulation syndrome, pregnancy

## Abstract

**Objective:**

To investigate whether letrozole priming could be used efficiently in patients undergoing *in vitro* maturation (IVM) as compared with follicle-stimulating hormone (FSH) priming.

**Materials and Methods:**

This is a retrospective analysis of 63 patients who underwent IVM due to the high risk of Ovarian Hyperstimulation syndrome (OHSS) (n=39), cancerophobia (n=16), and desire for IVM after failed *in vitro* fertilization attempts (n=8). Forty-two patients received FSH priming and 21 patients received letrozole priming.

**Results:**

The patients who had FSH or letrozole priming were statistically similar with respect to age, body mass index, duration of infertility, basal antral follicle count, serum anti-Müllerian hormone levels, and IVM indications (p>0.05 for all). When compared with the FSH priming group, the number of germinal vesicle oocytes, metaphase II and fertilized oocytes were significantly higher (p=0.003, p=0.001, and p=0.016, respectively), but the number of metaphase I oocytes was significantly lower in the letrozole priming group (p=0.002). The patients who received FSH and letrozole priming had statistically similar rates of implantation (33.3% vs 37.0%, p=0.709), clinical pregnancy (31.5% vs 33.3%, p=0.848), twinning (1.9% vs 3.7%, p=0.611), and live birth (24.1% vs 29.6%, p=0.682).

**Conclusion:**

Potential indications for IVM include patients with increased risk for OHSS and contraindication for hyperestrogenism. Aromatase inhibitors can be used to preserve the fertility of patients with estrogen-sensitive cancers. Letrozole priming appears to be an efficient approach in patients who undergo IVM, with likely less cost than FSH priming.

**PRECIS:** Letrozole priming *in vitro* maturation protocol has promising results in cases with cancerphobia and patients with history of OHSS. The outcomes are comparable with FSH priming IVM.

## Introduction

*In vitro* maturation (IVM) refers to the retrieval of immature oocytes from antral follicles, with the final stages of meiosis completed during *invitro* culture^(1)^. Potential indications for IVM include patients with increased risk for Ovarian Hyperstimulation syndrome (OHSS), patients with limited time for ovarian stimulation, and patients with a contraindication for elevated concentrations of estradiol^(1,2,3)^.

The primary benefits of IVM are the reduction in gonadotropin exposure, the subsequent decrease in the risk of OHSS, and facilitation of oocyte retrieval in oncology patients who need to undergo gonadotoxic treatment without adequate time for ovarian stimulation^(3,4)^. Another benefit of IVM is the avoidance from ovarian stimulation-related hyperestrogenism in oncology patients with hormone-sensitive tumors^(4,5)^.

Letrozole is an aromatase inhibitor that blocks the conversion of androgens into estrogens in the ovarian milieu^(6)^. Letrozole exerts its primary action by increasing endogenous secretion of follicle-stimulating hormone (FSH) and a secondary action by giving rise to a hyperandrogenic microenvironment, which triggers the development of primordial follicles^(7)^. Therefore, it has been hypothesized that letrozole could be used to achieve ovarian stimulation in women with hormone-sensitive tumors without exposing them to sustained elevated estrogen levels^(8)^. The avoidance from hyperestrogenism might also help to relieve the anxiety of infertile women who might be concerned about the long-term probability of developing estrogen-sensitive cancers while they are undergoing an assisted reproductive cycle^(8,9,10)^. On the other hand, the emergence of a hyperandrogenic microenvironment might be beneficial in cases of oocyte maturation arrest^(11,12)^.

This study aimed to investigate whether letrozole priming could be used efficiently for patients who were to undergo IVM treatment due to a high risk of OHSS, desire for IVM or fear for estrogen-sensitive cancers.

## Materials and Methods

This is a retrospective analysis of 63 patients who underwent IVM treatment due to the high risk of OHSS (n=39), fear for estrogen-sensitive cancers (n=16), and desire IVM after IVF failure (n=8) at Samsun Medicana International Hospital between September 2017 and January 2020. The primary risk factors for OHSS included young age (<33 years), polycystic ovaries on transvaginal ultrasonography (>24 antral follicles), and previous OHSS^(13)^. The women with cancerophobia were those with Polycystic Ovary syndrome (PCOS) who had fibrocystic breast disease (n=10), a family history of breast cancer (n=3), and family history of endometrium cancer (n=3). This study was approved by the Institutional Review Board of the study center (approval no: 3/2020) and conducted in accordance with the ethical principles outlined in the Declaration of Helsinki. All participants gave written informed consent.

Forty-two patients received FSH- human chorionic gonadotropin (hCG) priming and 21 patients received letrozole-hCG priming. Cancerophobia in the letrozole priming group was related to fibrocystic breast disease (n=5), positive family history of breast cancer (n=1) or endometrial cancer (n=1). Patients with hyperprolactinemia, Cushing’s syndrome, non-classic congenital adrenal hyperplasia, adrenal and ovarian androgen-secreting tumors were excluded. Women whose partners had azoospermia, cryptozoospermia, and severe oligoasthenoteratozoospermia were also excluded.

### Gonadotropin and Letrozole Priming

The standard protocol for IVM indicated FSH-hCG priming at the study center, as previously described^(14)^. IVM with letrozole priming was used only for the aforementioned indications. Beginning on the 3^rd^ day of a spontaneous or an induced menstrual cycle, 21 patients had a daily dose of 5 mg letrozole (Femara^®^, Novartis, Basel, Switzerland) for 3 days, and 42 patients received recombinant FSH (Gonal-f^®^, Merck-Serono, Geneva, Switzerland) at a daily dosage of 75 IU for 3 days. Either 10,000 IU of urinary hCG (Pregnyl^®^, Organon, Amsterdam, Netherlands) or 250 µg recombinant hCG (Ovitrelle^®^, Serono, Geneva, Switzerland) was administered when the leading follicle size was 10 to 12 mm and endometrial thickness was at least 8 mm on transvaginal ultrasonography in the mid-sagittal plane. Under the guidance of transvaginal ultrasonography, oocyte pickup was performed using a 17-gauge double-lumen aspiration needle with an aspiration pressure of 80 mmHg 36-38 hours after hCG administration.

### *In Vitro* Maturation

Follicular aspirates were collected into 14 mL Falcon tubes (Falcon, Code 352001), which were kept at 37 °C and filtered using a strainer (Falcon-70 µm pore size-, Code 352350). Under a stereomicroscope (Olympus SZ 61, Shibuya, Tokyo, Japan), oocytes were isolated into the perimeter of a Falcon center-well dish containing flushing medium without heparin and subsequently incubated for two to three hours in IVM media.

Upon completion of oocyte collection, the final medium was prepared using commercially available IVM medium (Vial 2 Medi-Cult IVM System), 0.5 mL of patient serum, 50 µL FSH prepared from a stock solution of 0.075 IU/mL (75 IU GONAL-f was diluted with 10 mL IVM medium and 5 µL hCG, and then incubated at 37 °C and 6% CO_2_ and 5% O_2_. After incubation in LAG medium, oocytes were placed equally in the wells of a four-well dish (Nunc, Roskilde, Denmark) containing 0.6 mL of final IVM medium covered with liquid paraffin and incubated for 26-28 hours. Nuclear maturity of oocytes was not assessed any further.

### Intracytoplasmic Sperm Injection

Intracytoplasmic sperm injection (ICSI) was performed in all IVM cycles. The spermatozoa were prepared using a three-layer PureSperm gradient (Codes PSB 100 and PS100-100, Mölndal, Gothenburg, Sweden). Removal of the cumulus and corona cells was performed in a hyaluronidase-containing medium using Pasteur pipettes after a 26-28 h incubation period. The oocytes were then transferred to U-IVF medium for culture. All ICSI procedures were performed in Falcon Petri dish with droplets of PVP containing medium for sperms (Vitrolife, Code 10111, 5x0.1 mL) and droplets of flushing medium without heparin for oocytes.

When the ICSI procedure was completed, the oocytes were placed into ISM 1 medium for culture. After the oocyte fertilization was evaluated in droplets of ISM 1 medium, incubation of fertilized oocytes was continued for 24-hours (which made up 48-hours in total). The first fertilization check, which occurred 16-18 h after ICSI was determined by the presence of two distinct pronuclei and two polar bodies within the oocytes in ISM 1 medium through an inverted microscope at x200 magnification. This was repeated 24 hours later.

The classification system introduced by Veeck was used for the evaluation of embryo grade on the third day of culture. The embryos were graded as follows: Grade 1 - embryo with blastomeres of equal size, no cytoplasmic fragments; Grade 2 - embryo with blastomeres of equal size, minor cytoplasmic fragments or blebs: Grade 3 - embryo with blastomeres of distinctly unequal size, none or few cytoplasmic fragments^(15)^.

Embryo transfer was performed using a Wallace embryo transfer catheter (Wallace, UK) under abdominal ultrasonography guidance and placed 1.5 to 2.5 cm from the fundus. Women aged younger than 35 years could only have a single embryo transferred, and a maximum of two embryos could be transferred to women aged over 35 years. Taking endometrial thickness into consideration, fresh embryo transfers were performed on day 3 or day 5. To prepare the endometrium for embryo transfer, an oral estrogen tablet was administered at a daily dosage of 4 mg (Estrofem^®^, Novo Nordisk, Bagsværd, Denmark) beginning from the third day of menstrual cycle. Luteal phase support was begun the day following OPU, which was provided with a vaginal progesterone capsule at a daily dosage of 200 mg (Progestan^®^, Koçak Farma, İstanbul, Turkey). Estrogen tablets and progesterone capsules were continued until the first fetal heartbeat was detected.

Clinical pregnancy was defined as the presence of at least one gestational sac in the uterus. No IVM cycles were cancelled in this study.

### Statistical Analysis

Collected data were analyzed using the Statistical Package for the Social Sciences version 22.0 software (SPSS IBM Inc., Armonk, NY, USA). Continuous variables are expressed as mean ± standard deviation and categorical variables are denoted as numbers or percentages. Continuous variables were compared using Student’s t-test or the Mann-Whitney U test, whereas categorical variables were compared using the chi-square test. Two-tailed p values ≤0.05 were regarded as statistically significant. A post-hoc analysis was performed to determine that a cohort size of 63 women (undergoing IVM for minimizing OHSS risk and preventing oocytes maturation arrest) had 67.6% power to detect a difference at the 0.05 significance level.

## Results

Table 1 compares the baseline characteristics of the 21 patients who had letrozole priming and 42 patients who received FSH priming for IVM treatment. There were no significant group differences in age, body mass index, duration of infertility, basal antral follicle count, and serum AMH concentrations (p>0.05 for all). The patients who underwent FSH and letrozole priming were also statistically similar in the aspect of IVM indications (Table 2).

Table 3 summarizes the laboratory findings of the patients. Both the FSH and letrozole priming groups were statistically similar concerning the endometrium thickness on the hCG day, time to oocyte pick up, retrieved oocytes counts, and single or double embryo transfers (p>0.05 for all). When compared with the FSH priming group, a significantly higher number of germinal vesicle oocytes, metaphase II, and fertilized oocytes (p=0.003, p=0.001, p=0.016, respectively) and a significantly lower number of metaphase I oocytes and grade 3 embyros were detected in the letrozole priming group (p=0.002, p=0.007, respectively). The number of three-cell embryos was also significantly lower in the letrozole priming group when compared with the FSH priming group (p=0.007).

Table 4 shows the pregnancy outcomes of the patients. The patients who received FSH and letrozole priming had statistically similar rates of implantation (33.3% vs 37%, p=0.709), clinical pregnancy (31.5% vs 33.3%, p=0.848), twin pregnancy (1.9% vs 3.7%, p=0.611), and live birth (24.1% vs 29.6%, p=0.682).

## Discussion

By definition, IVM refers to the maturation of collected immature oocytes under the influence of hormones that exist within the culture media. Thus, hormones are not administered to patients undergoing IVF treatment^(16,17)^. However, the general opinion about IVM is that a short priming protocol with FSH stimulation increases the chance for oocyte maturation and implantation in patients with PCOS. This short priming protocol involves the administration of FSH at a dose of 75 IU to 150 IU for a period of two to five days. FSH stimulation at the beginning of a cycle increases the number of immature oocytes retrieved and thus clinical pregnancy rates in IVM cycles^(18,19,20)^. Accordingly, this study adopted an IVM protocol indicating the administration of recombinant FSH at a dose of 75 IU for three days.

In this study, 16 patients with PCOS refused to receive FSH priming because they had cancerophobia due to fibrocystic breast disease and a positive family history for breast and endometrium cancer. Therefore, priming was performed using an aromatase inhibitor, namely letrozole, in seven patients with PCOS.

In this study, the primary indications for IVM treatment were the avoidance from OHSS and previously failed IVF desiring IVM. It is well known that IVM is the only assisted reproduction technique that has been proved to be devoid of OHSS risk. Recent meta-analyses reported that patients treated with IVM had significantly higher implantation and clinical pregnancy rates, as well as significantly lower miscarriage and cycle cancellation rates^(22,23)^. The maturation of human oocytes is naturally arrested at the germinal vesicle stage when the oocytes need gonadotropin stimulation and the metaphase II stage when oocytes are waiting for fertilization. A therapeutic approach for failed IVF could be the IVM of immature oocytes within culture media enriched with factors necessary for oocyte maturation^(11,12)^, if compromised *in vivo*.

Due to the wide variations in priming protocols, varying numbers between 8% and 40% have been specified as the clinical pregnancy rates and live birth rates in IVM cycles^(24)^. Similarly, our previous study reported clinical pregnancy rates of 44.6% and 44.7% and live birth rates of 34.9% and 34.2% for single-embryo transfer and double-embryo transfers in IVM cycles with FSH priming^(14)^. As for the present study, the clinical pregnancy and live birth rates were respectively 31.5% and 24.1% in IVM cycles with gonadotropin priming. Although clinical pregnancy and live birth rates tended to be higher in IVM cycles with letrozole priming (33.3% and 29.6%), there were no statistically significant differences.

A new indication for IVM has been defined as a convenient therapeutic approach for infertile patients who have been diagnosed as having malignancies and scheduled for oncofertility treatment. The basic rationale for this definition is the feasibility of IVM for preserving oocytes and embryos and thus, future fertility, whenever conventional *in vitro* fertilization is not an option. A potential factor that might delay the oncology treatment can also be avoided because IVM prevents the risk of OHSS. Additionally, this technique can be used to replace an IVF cycle that would otherwise end up with excessive follicular growth and subsequent cycle cancellation^(25,26)^.

Another advantage of IVM treatment is that it can be started immediately at any time of the menstrual cycle without stimulating ovaries. Therefore, IVM emerges as an appropriate technique for preserving the fertility of oncology patients in whom the initiation of chemotherapy, radiotherapy or surgical treatment cannot be delayed^(1,2)^. Another advantage of this assisted reproduction technique is that serum estrogen concentrations remain low throughout an IVM cycle. Therefore, IVM has been established as a safe and effective method for fertility preservation in patients with estrogen-sensitive tumors^(25,26)^.

It is also well known that aromatase inhibitors can be used as single agents or adjuncts to FSH-containing ovulation induction regimens for preserving the fertility of patients with estrogen-sensitive breast cancer. Aromatase inhibitors would reduce supra-physiologically serum concentrations of estradiol, suppress local production of estrogen within the tumor tissue, and induce follicular growth in women with estrogen-sensitive malignancies. Combining gonadotropins with aromatase inhibitors would augment the ovarian stimulation without a profound increase in serum estradiol levels^(27,28)^.

## Conclusion

The findings of this study suggest that IVM treatment with letrozole priming might be an efficient approach for patients who have a high risk for OHSS or fear for estrogen-sensitive tumors. However, these findings should be interpreted carefully as their power is limited by its retrospective design, small cohort size, technical inadequacy for cryopreservation, and lack of long-term data. Further research is warranted to clarify the clinical implications of letrozole priming in IVM cycles. A prospective study of letrozole-primed IVM should be considered.

## Figures and Tables

**Table 1:**
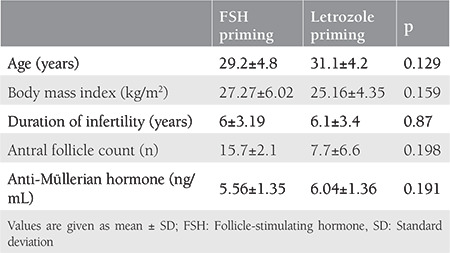
Baseline characteristics of the patients

**Table 2:**
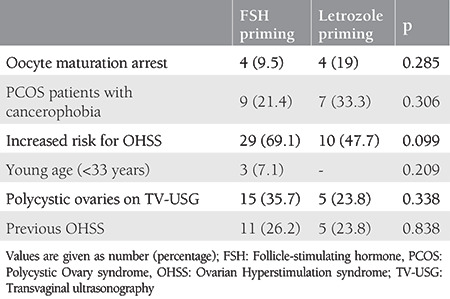
Indications for in vitro maturation

**Table 3:**
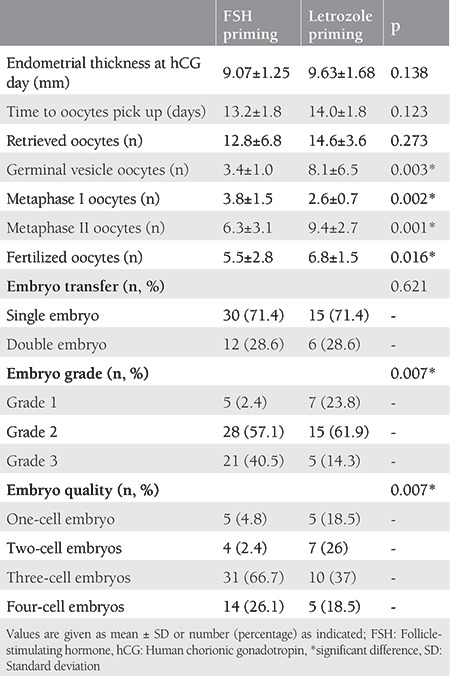
Laboratory findings of the patients

**Table 4:**
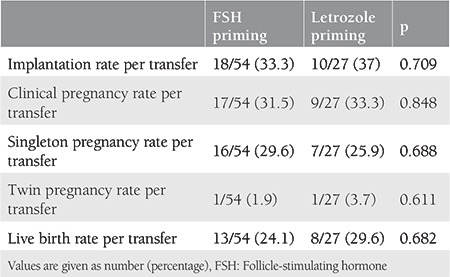
Comparison of pregnancy outcomes of two groups
